# 3D Electron-Rich ZIF-67 Coordination Compounds Based on 2-Methylimidazole: Synthesis, Characterization and Effect on Thermal Decomposition of RDX, HMX, CL-20, DAP-4 and AP

**DOI:** 10.3390/molecules27238370

**Published:** 2022-11-30

**Authors:** Xiong Yang, Bojun Tan, Bo Wang, Lina Yao, Xin Li, Dongkui Zhao, Wenjie Li, Lei Cao, Yafeng Huang, Xiaofeng Wang

**Affiliations:** Xi’an Modern Chemistry Research Institute, Xi’an 710065, China

**Keywords:** solid propellant, energetic metal organic frameworks (EMOFs), zeolite imidazolate frameworks-67 (ZIF-67), energetic burning rate catalysts, burning rate catalysts

## Abstract

ZIF-67 is a three-dimensional zeolite imidazole ester framework material with a porous rhombic dodecahedral structure, a large specific surface area and excellent thermal stability. In this paper, the catalytic effect of ZIF-67 on five kinds of energetic materials, including RDX, HMX, CL-20, AP and the new heat-resistant energetic compound DAP-4, was investigated. It was found that when the mass fraction of ZIF-67 was 2%, it showed excellent performance in catalyzing the said compounds. Specifically, ZIF-67 reduced the thermal decomposition peak temperatures of RDX, HMX, CL-20 and DAP-4 by 22.3 °C, 18.8 °C, 4.7 °C and 10.5 °C, respectively. In addition, ZIF-67 lowered the low-temperature and high-temperature thermal decomposition peak temperatures of AP by 27.1 °C and 82.3 °C, respectively. Excitingly, after the addition of ZIF-67, the thermal decomposition temperature of the new heat-resistant high explosive DAP-4 declined by approximately 10.5 °C. In addition, the kinetic parameters of the RDX+ZIF-67, HMX+ZIF-67, CL-20+ZIF-67 and DAP-4+ZIF-67 compounds were analyzed. After the addition of the ZIF-67 catalyst, the activation energy of the four energetic materials decreased, especially HMX+ZIF-67, whose activation energy was approximately 190 kJ·mol^−1^ lower than that reported previously for HMX. Finally, the catalytic mechanism of ZIF-67 was summarized. ZIF-67 is a potential lead-free, green, insensitive and universal EMOFs-based energetic burning rate catalyst with a bright prospect for application in solid propellants in the future.

## 1. Introduction

Technologies that can regulate the combustion performance of solid propellants have always been the focus of research on solid propellant applications. The burning rate dictates the amount of fuel gas generated by the propellant, the motor thrust and the burning area of the thrust. The burning rate catalyst is an indispensable functional material component that can effectively regulate the burning rate of solid propellants, reduce the pressure index, and thereby adjust and improve the burning performance of solid propellants [1,2,3]. Metal, metal oxide and metal composite oxide catalysts are earlier used and thoroughly studied burning rate catalysts, which have good catalytic effects on most solid propellants. Current research on such catalysts mainly focuses on the preparation of nanoscale catalysts with exceptional catalytic performance from effective metals or metal oxides [4,5,6,7]. However, nano-sized catalysts have two shortcomings. On the one hand, nanoscale metal oxide and composite metal oxide catalysts have a small specific surface area and low catalytic activity. The reason is that these two types of catalysts often need to be calcined, or they absorb other substances when exposed to air in the preparation process. On the other hand, the inert metal or composite metal oxide raw materials lower the energy level of solid propellants [8,9].

**Figure 1:**
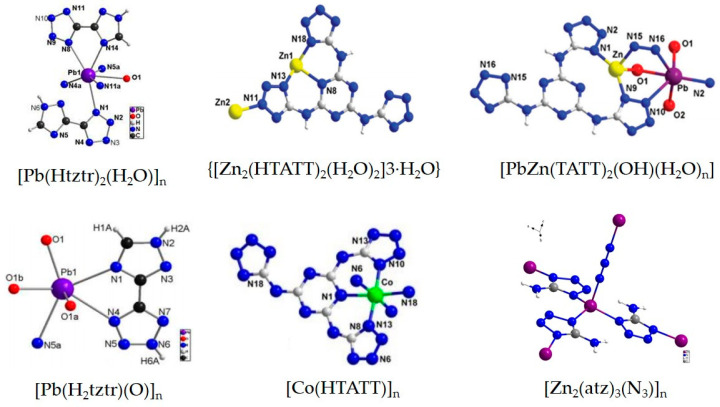
The structure diagram for the EMOFs mentioned above.

In order to solve the above problems, researchers have studied energetic metal-organic framework (EMOFs)-based energetic burning rate catalysts in recent years. EMOF-based energetic burning rate catalysts contain a large number of N=N and C-N bonds with a high formation enthalpy, and they have a larger density, higher oxygen balance, and more gas production per unit mass than ordinary substances. Therefore, such catalysts have a higher formation enthalpy and higher energy. Their catalytic principle is to produce fresh nanoscale or microscale metal oxides or metal composite oxides in situ during the combustion and decomposition of solid propellants. This method overcomes the problem of the preparation method or product agglomeration degrading the catalytic performance of ordinary nanoscale or microscale catalysts, and it greatly improves the catalytic performance of the energetic catalysts. Energetic catalysts are a new type of energetic burning rate catalyst with both the advantages of high energy and a nano size, having great potential advantages in solid propellant applications and requiring vigorous development. Chen et al. [10] prepared two EMOFs-based energetic burning rate catalysts, [Pb(Htztr)_2_(H_2_O)]_n_ and [Pb(H_2_tztr)(O)]_n_, through the coordination reaction between lead nitrate and the energy ligand 5-tetrazolyl-1,2,4-triazole (H_2_tztr). The catalytic pyrolysis experiment showed that [Pb(Htztr)_2_(H_2_O)]_n_ catalyzed the two exothermic peaks of pure ammonium perchlorate (AP) into one violent exothermic peak and reduced the exothermic peak temperature by approximately 19 °C. Additionally, [Pb(H_2_tztr)(O)]_n_ lowered the exothermic peak temperature of AP by approximately 18 °C. In addition, [Pb(Htztr)_2_(H_2_O)]_n_ and [Pb(H_2_tztr)(O)]_n_ reduced the exothermic peak of hexahydro-1,3,5-trinitro-1,3,5-triazine (RDX) by 16 °C and 11 °C, respectively. In the research of Wu et al. [11], the nitrogen-rich Y-shaped high-energy ligand 3-(5-aminotetrazole) triazine (H_3_TATT) in solvent H_2_O reacted with CoCl_2_·6H_2_O under hydrothermal conditions to synthesize a solvent-free energetic metal organic framework (EMOF) [Co(HTATT)]_n_. The complex obtained had good thermal stability, low impact sensitivity and friction sensitivity. Compared with the pure AP component, the mixture of [Co(HTATT)]_n_ and AP converted the two exothermic peaks of AP into a larger exothermic peak with a greatly decreased peak temperature of 326 °C and sharply increased the decomposition heat of 2.39 kJ·g^−1^ (increase rate: 300%). Wu et al. [12] coordinated the energetic ligand H_3_TATT with Zn(II) and Pb(II) to prepare two energetic complexes, namely, {[Zn_2_(HTATT)_2_(H_2_O)_2_]_3_·H_2_O} and [PbZn(TATT)_2_(OH)(H_2_O)_n_]. Both of them could accelerate the thermal decomposition of RDX as burning rate catalysts. [PbZn(TATT)_2_(OH)(H_2_O)_n_] reduced the decomposition temperature by approximately 25 °C. In 2022, Tan et al. [13] developed a two-dimensional azide bridged energetic metal organic framework material [Zn_2_(atz)_3_(N_3_)]_n_ by coordinating the ligand 5-aminotetrazole(atz) with zinc ions. This complex showed excellent heat resistance (thermal decomposition temperature was 362 °C) and low sensitivity, and it reduced the thermal decomposition temperatures of AP and 2,4,6,8,10,12-hexanitro-2,4,6,8,10,12-hexaazaisowurtzitane (CL-20) by 50 °C and 6 °C, respectively. The structure of the above EMOFs is shown in Figure 1.

**Figure 2:**
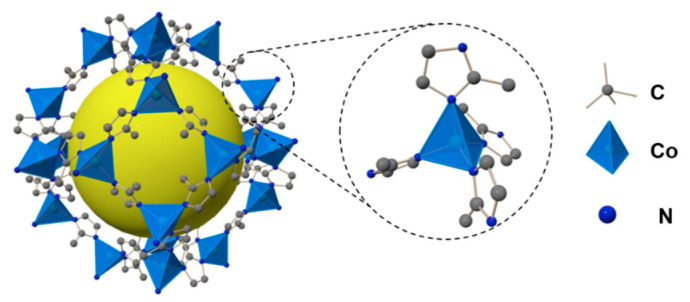
A structure diagram of ZIF-67-based EMOFs.

EMOFs-based energetic burning rate catalysts have been reported to have excellent catalytic effects on specific solid propellant formulations. Existing solid propellants have diversified formulations and complex components, but EMOFs-based energetic burning rate catalysts are selective and can only catalyze one or two propellant components. They have almost no catalytic effect on other types of propellant components [7]. The type and structure of catalysts are the main factors affecting the catalytic effect. A new type of catalyst is required for a new solid propellant formulation. Therefore, there are plans to introduce an energetic burning rate catalyst with universal performance into the field of solid propellants. This catalyst should exhibit excellent catalytic effects on multiple types of solid propellant components. After reviewing a large quantity of literature, zeolite imidazolate frameworks-67 (ZIF-67), reported on by Daren Chen’s research group [14] in 2013, were found to be a likely candidate for an EMOFs-based energetic burning rate catalyst with great application potential and universal performance. ZIF-67 is a three-dimensional zeolite imidazole ester framework material with a porous rhombic dodecahedral structure (Figure 2), a large specific surface area (1500 m^2^·g^−1^) and high thermal stability (a thermal decomposition temperature greater than 450 °C), but there is no relevant study on the application of ZIF-67 in catalyzing solid propellants currently. To fill this gap in the research, this paper introduces ZIF-67 as a new type of EMOFs-based energetic burning rate catalyst into the field of solid propellants. It was found that 2 wt% ZIF-67 could catalyze five energetic materials well. Under the same test conditions (10 °C·min^−1^), 2 wt% ZIF-67 tremendously decreased the thermal decomposition temperatures of RDX, 1,3,5,7-tetranitro-1,3,5,7-tetraazacyclooctane (HMX), CL-20 and AP, which are the main energetic components of solid propellants. Specifically, 2 wt% ZIF-67 reduced the thermal decomposition peak temperatures of RDX, HMX and CL-20 by 22.3 °C, 18.8 °C and 4.7 °C, respectively. The low- and high-temperature thermal decomposition peak temperatures of AP declined by 27.1 °C and 82.3 °C, respectively, after the addition of 2 wt % ZIF-67. Moreover, 2 wt% ZIF-67 also exhibited excellent catalytic effects on the recently developed subversive heat-resistant energetic material (H_2_dabco) [NH_4_ (ClO_4_)_3_] (DAP-4) [15,16,17]. It reduced the thermal decomposition temperature of DAP-4 by approximately 10.1 °C. To sum up, ZIF-67 shows excellent universal catalytic effects on ammonium nitrate and salt energetic materials.

## 2. Results and Discussion

### 2.1. Synthesis of ZIF-67

**Scheme 1:**
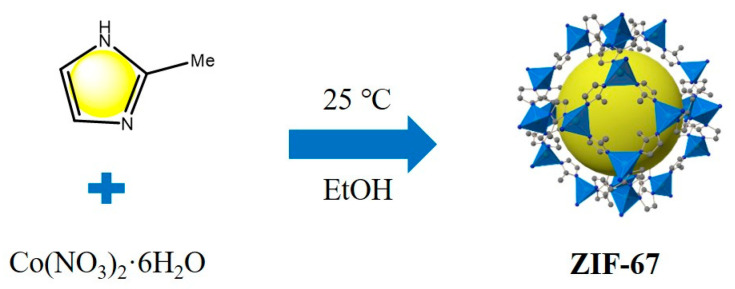
Schematic diagram for ZIF-67 synthesis.

ZIF-67 (Scheme 1) was prepared by a simple process. First, Co(NO_3_)_2_·6H_2_O (1 mmol, 249.0 mg) and 2-methylimidazole (4 mmol, 328.0 mg) were dissolved in 25 mL ethanol separately. After stirring and mixing the two solutions at 25 °C for 0.5 h, the resultant solution was precipitated for 24 h. Finally, the precipitate was centrifuged to separate the washing product. The separation yield was 86%. When increasing the feed amount of the materials by 10 times and preparing ZIF-67 in the same reaction conditions, the separation yield was 78%. In the case of poor dispersibility during the preparation process, the materials were fully stirred or ultrasonically treated until they were completely dispersed in the solution.

### 2.2. Characterization of ZIF-67

**Figure 3:**
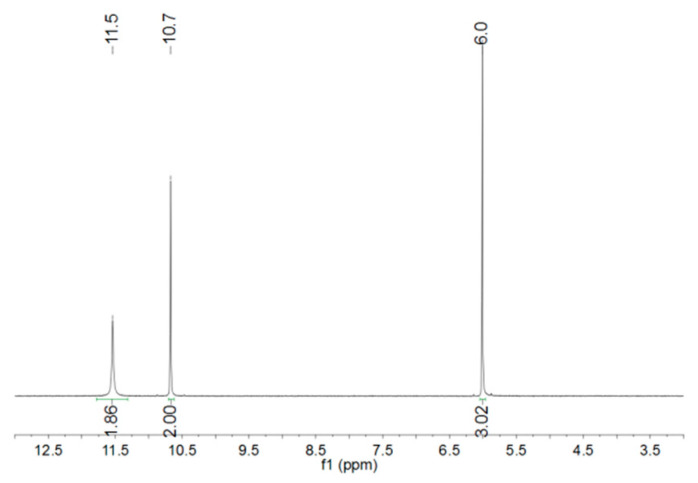
The ^1^HNMR spectrogram (in DCl).

**Figure 4:**
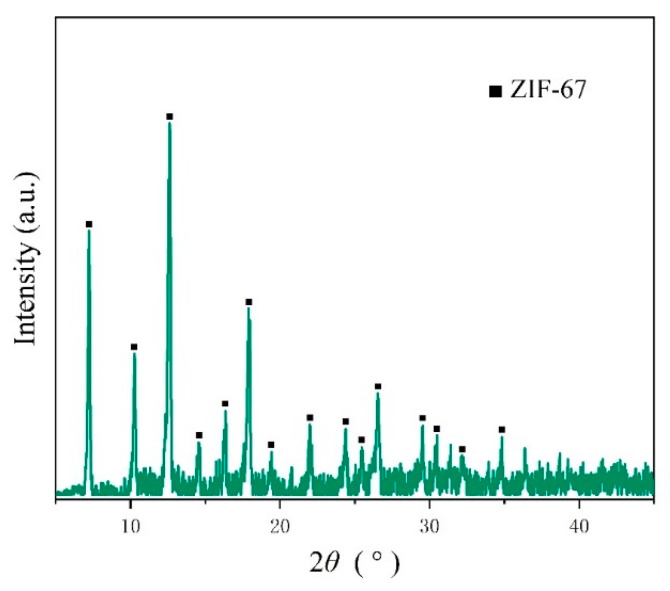
The XRD spectrogram.

**Figure 5:**
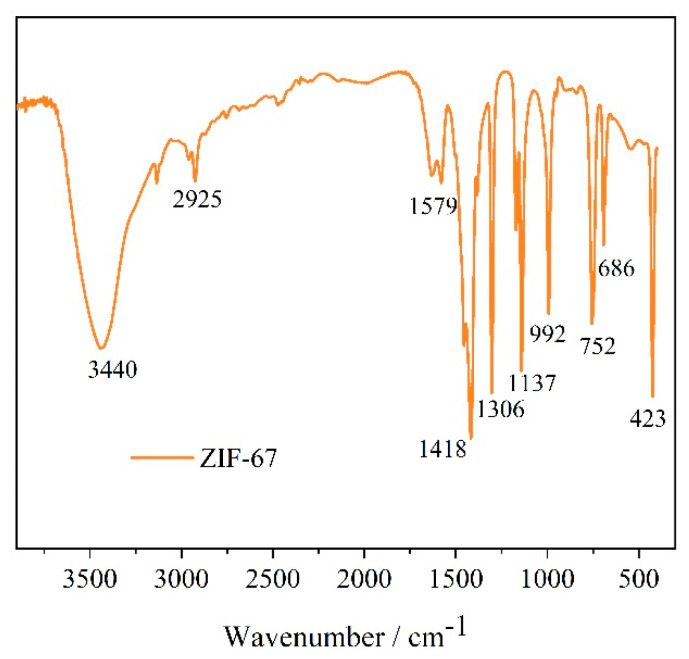
The IR spectrogram.

The structure of ZIF-67 was characterized by ^1^HNMR, X-ray diffraction and Fourier transform infrared technology. The correlation spectrograms for these are found in Figure 3, Figure 4 and Figure 5. It can be seen from the XRD spectra that the 2θ of ZIF-67 had Strong diffraction peaks appearing at 7.38°, 10.42°, 12.74° and 18.06°, which are consistent with the positions of the ZIF-67 characteristic peaks reported in the literature [18]. In the infrared spectrum, the peak at 2925 cm^−1^ is the asymmetric vibration peak for the C-H bond in CH_3_ group; the absorption peak at 1579 cm^−1^ is the stretching vibration peak of C=N bond; the peaks at 500–1500 cm^−1^ are the plane bending vibration peaks and stretching vibration peaks of imidazole ring; and the peak at 752 cm^−1^ is the stretching vibration peak of the Co-N bond. The positions of the peaks are consistent with the literature [19].

Mechanical sensitivity performance is an important safety performance index for energetic materials, and friction sensitivity and impact sensitivity are the two most representative safety performance indexes. In order to evaluate the safety of the ZIF-67 application in propellant formulations, friction sensitivity and impact sensitivity tests were conducted [13]. In the impact sensitivity test, a hammer with a weight of 2 kg was dropped from a set height onto a sample with a weight of 20 mg at 39.2 MPa. The height (H_50_) represented the drop height with a 50% ignition probability. The final results showed that ZIF-67 did not ignite at the highest position of 200 cm, which corresponded to 40 J impact energy. At the same time, the friction sensitivity of a 20 mg sample weight was measured by using the Julius Peter device. The measurement results suggested that no reaction occurred below 360 N. Taken above, ZIF-67 has excellent mechanical sensitivity safety and can be used as an additive component of solid propellant formulations.

### 2.3. Thermal Decomposition of RDX, HMX, CL-20, DAP-4 and AP Catalyzed by ZIF-67

**Figure 6:**
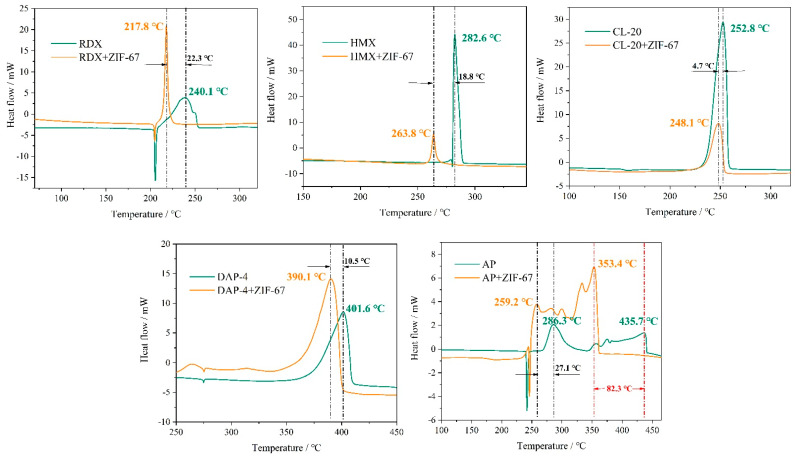
DSC curves for the mixtures of ZIF-67 and five different energetic materials.

In order to explore the catalytic effect of ZIF-67 on the thermal decomposition of RDX, HMX, CL-20, DAP-4 and AP, along with 2 wt% of ZIF-67 was added to the five energetic materials, and the mixtures were ground for more than 30 minutes to enable them to mix fully. The DSC curves of five composite energetic materials heated from room temperature to 450 °C at a heating rate of 10 °C·min^−1^ were tested, and the results are shown in Figure 6. It can be seen from the DSC curves that ZIF-67 reduced the thermal decomposition peak temperatures of RDX, HMX, CL-20 and DAP-4 by 22.3 °C, 18.8 °C, 4.7 °C and 10.5 °C, respectively. After the addition of ZIF-67, the low-temperature and high-temperature thermal decomposition peak temperatures of AP decreased by 27.1 °C and 82.3 °C, respectively. The results show that ZIF-67 can effectively catalyze the thermal decomposition of the above five energetic materials and has good universal catalytic performance.

Through our analysis, we believe that ZIF-67-based EMOFs with regular pore structure and large specific surface area possess ab incomparable superiority relative to other burning rate catalysts for solid propellants. On the one hand, the large specific surface area promotes full contact between ZIF-67-based EMOFs and other components of the solid propellant, and different interactions between organic ligands and metal centers affect the structural evolution of catalysts during the combustion catalytic reaction. On the other hand, the regular pore structure facilitates a uniform particle size distribution of ZIF-67-based EMOFs, which can more easily change the decomposition process of the solid propellant in the sub surface reaction zone or on the combustion surface. ZIF-67-based EMOFs catalyze the solid propellant components homogeneously and synergistically at the molecular level, greatly accelerating their thermal decomposition. In this way, ZIF-67-added EMOFs achieve effective catalysis. In addition, the moderate carbon content in ZIF-67-based EMOFs makes the catalysts a catalytic bed for metal cobalt enrichment and helps to block cobalt condensation [20,21,22].

### 2.4. Thermal Decomposition Mechanism of Typical Energetic Materials HMX and HMX+ZIF-67

In order to prove that the reason why ZIF-67 advances the thermal decomposition temperature of the above energetic materials is the catalysis, we selected the typical energetic materials HMX and HMX+ZIF-67, tested and compared the heat release using DSC (10 K·min^−1^), and analyzed their thermal decomposition mechanism with a mass spectra (MS) test.

**Figure 7:**
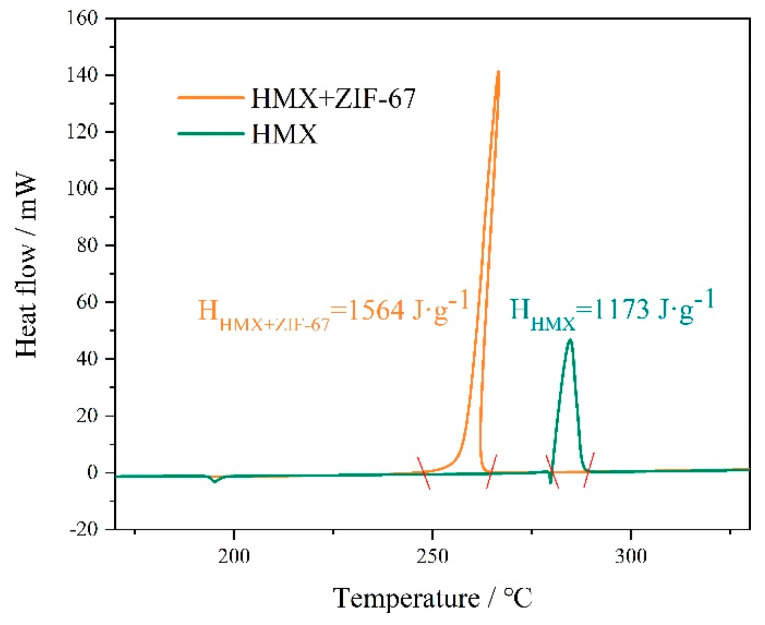
The heat release of HMX and HMX+ZIF-67.

The results of the experiment are showed in Figure 7. After adding the burning rate catalyst, the heat release of HMX can be increased from 1173 J·g^−1^ to even as high as 1564 J·g^−1^, which also indicates that ZIF-67, as a general burning rate catalyst, has a very excellent catalytic effect on HMX, which not only makes the decomposition temperature of HMX advance 18.8 °C, but also makes its heat release increase significantly.

**Figure 8:**
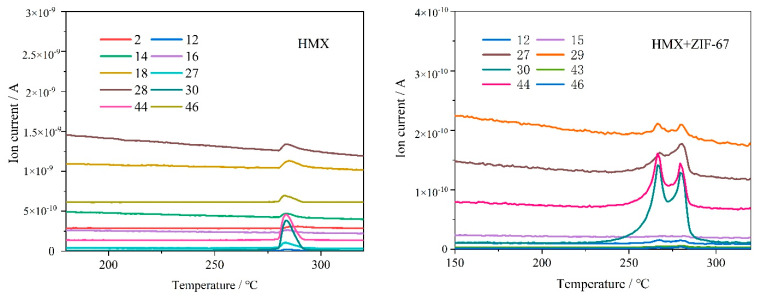
MS spectrogram for the products of HMX and HMX+ZIF-67.

The MS spectrogram for the products of HMXand HMX+ZIF-67 is shown in Figure 8. It can be seen from the mass spectra of HMX and HMX+ZIF-67 that the amount of fragments (*m*/*z* = 30 and *m*/*z* = 44) in both were the largest. A small amount of fragments of *m*/*z* = 30 were first detected in the MS of HMX and HMX+ZIF-67 at 277 °C or 235 °C, which indicates that a small amount of nitro and nitroso were transformed into each other in the process of the denitrication of HMX, and a small amount of NO (*m*/*z* = 30) was first decomposed and released [23,24]. A large number of *m*/*z* = 30 fragments in the mass spectrogram of HMX began to be produced at 280 °C. At the same time, a large amount of *m*/*z* = 44 fragments began to be released. The analysis shows that the substance corresponding to *m*/*z* = 30 should be mainly CH_2_O and the fragment corresponding to *m*/*z* = 44 is N_2_O, because the C-N bond on the HMX ring and the N-O bond in the nitro group begin to break at this time. In addition, we also found that a small number of fragments with *m*/*z* = 27 and *m*/*z* = 46 were formed at this time. It can be seen from the literature [25] that the thermal decomposition mechanism of HMX is that N-NO_2_ and C-N bond breaks at the same time. Therefore, the material fragments corresponding to *m*/*z* = 27 and *m*/*z* = 46 should be considered to be HCN and NO_2_, respectively.

However, we find that *m*/*z* = 30 fragments started to form at 235 °C in HMX+ZIF-67 and began to release in large quantities at 255 °C with a temperature span of 20 °C. The fragments of *m*/*z* = 30 started to form at 235 °C in HMX+ZIF-67 and began to release in large quantities at 255 °C with a temperature span of 20 °C. However, *m*/*z* = 30 fragments in the mass spectrogram of HMX started to generate at 277 °C and started to generate in large quantities at 280 °C, and the span was only 3 °C. This phenomenon strongly proves that ZIF-67 has a catalytic effect on HMX, catalyzes the exchange of nitro and nitroso, and causes the start temperature for NO release in the early stage of decomposition to be greatly advanced. In addition, fragments of *m*/*z* = 44 began to form at 245 °C, and a large number of fragments with *m*/*z* = 44 were released at 255 °C with a span of 10 °C. The fragments with *m*/*z* = 44 were directly generated in large quantities at 280 °C in HMX+ZIF-67 with a span of 0 °C, and there was no early slow-release process. This shows that ZIF-67 also has a catalytic effect on the splitting of the HMX ring.

In order to further compare the decomposition mechanism, we added some HMX into the sample cell containing HMX+ZIF-67. Surprisingly, there was a double peak phenomenon in the MS spectrum for the sample. We found that the first peak of MS=30 and MS = 44 was higher than the second peak, but the first peak of MS = 27 and MS = 46 was lower than the second peak. This phenomenon shows that, after adding ZIF-67, the catalysis promoted the formation of CH_2_O and N_2_O and inhibited the formation of HCN and NO_2_.

**Scheme 2:**
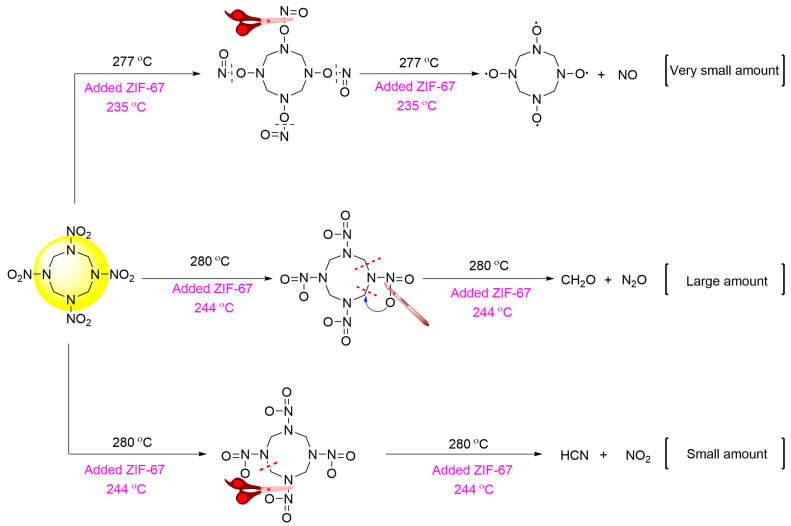
The thermal decomposition mechanism for HMX and HMX+ZIF-67.

The thermal decomposition mechanism for HMX and HMX+ZIF-67 is shown in Scheme 2.

### 2.5. Thermal Decomposition Kinetics of RDX, HMX, CL-20 and DAP-4 under ZIF-67 Catalytic Conditions

**Figure 9:**
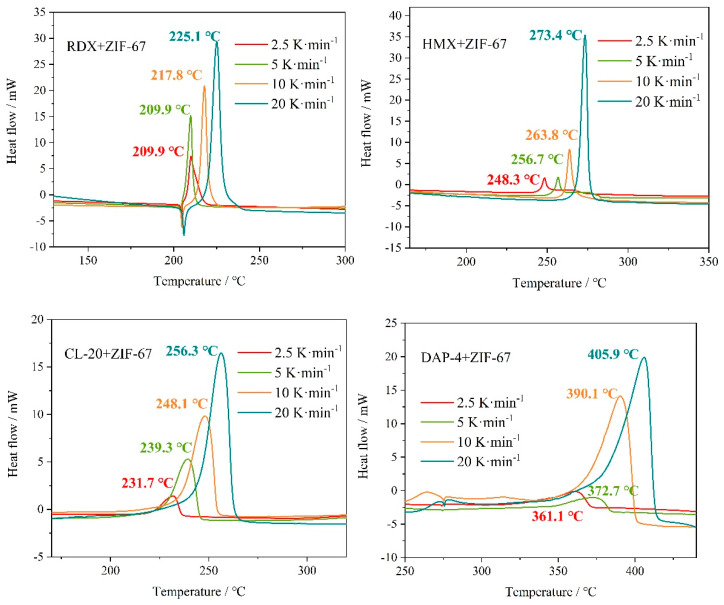
DSC curves for RDX, HMX, CL-20 and DAP-4 under ZIF-67 catalytic conditions at different heating rates.

The thermal behavior of RDX, HMX, CL-20 and DAP-4 under the catalysis of ZIF-67 was studied using non-isothermal DSC (Figure 9). The decomposition peak temperatures of RDX+ZIF-67, HMX+ZIF-67, CL-20+ZIF-67 and DAP-4+ZIF-67 at the heating rates of 2.5 K·min^−1^, 5 K·min^−1^, 10 K·min^−1^ and 20 K·min^−1^ are [209.9 °C, 209.9 °C, 217.8 °C and 225.1 °C], [248.3 °C, 256.7 °C, 263.8 °C and 273.4 °C], [231.7 °C, 239.3 °C, 248.1 °C and 256.3 °C], and [361.1 °C, 372.7 °C, 390.1 °C and 405.9 °C], respectively. It can be seen that the decomposition peaks of HMX+ZIF-67, CL-20+ZIF-67 and DAP-4+ZIF-67 move to the right with the increasing heating rate. However, the decomposition peak temperatures for RDX+ZIF-67 at the heating rates of 2.5 K·min^−1^ and 5 K·min^−1^ are the same. This abnormality indicates that the RDX-catalyzing efficiency of ZIF has reached its extreme value when the heating rate is as low as 5 K·min^−1^.

To investigate the effects of the ZIF-67 catalyst on the thermal decomposition kinetics of energetic materials, the kinetic parameters of RDX+ZIF-67, HMX+ZIF-67, CL-20+ZIF-67 and DAP-4+ZIF-67 were calculated by Kissinger, Friedman isoconversional and combined kinetic methods [26,27,28,29]. The results are listed in Table 1. The Ea values calculated by the Friedman method and the combined kinetic method were relatively close, but greatly differed from those obtained by the Kissinger method. Since the Kissinger method only takes the peak value into account and ignores the whole reaction process, large errors are prone to occur in the calculation process. According to the relevant literature, the data from the combined kinetic method are more reliable [29,30].

Previous studies have shown that the activation energy of RDX, HMX, CL-20 and DAP-4 is 211.7 kJ·mol^−1^ [31], 383.6 kJ·mol^−1^ [32], 238.6 kJ·mol^−1^ [32] and 205.4 kJ·mol^−1^ [33], respectively. Generally, higher activation energy indicates that more energy is required to reach the activation energy barrier, and it takes longer time for the decomposition reaction to initiate [34]. In this study, we found through comparison that the activation energy of the four energetic materials was reduced to varying degrees after the addition of ZIF-67. HMX+ZIF-67 had the largest decrease in activation energy, which was approximately 190 kJ·mol^−1^ lower than that reported previously for HMX.

**Figure 10:**
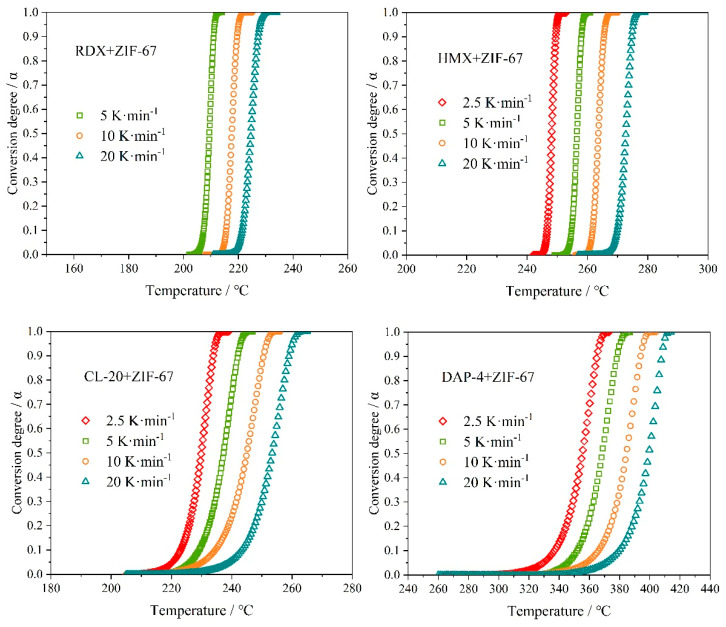
The α-T relationships of four composites.

**Figure 11:**
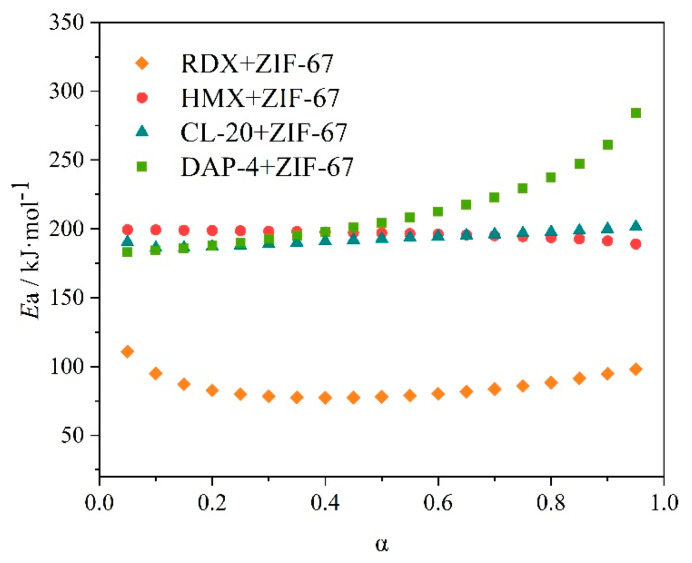
Ea-α relationships of four composites during the thermal decomposition process.

The Fraser–Suzuki equation [35] was used to fit the DSC decomposition exothermic peak from the original data in the Friedman method. The results revealed that all the correlation coefficients were greater than 0.98, which meets the accuracy requirements for kinetic evaluation. The conversion degree (α) of the temperature point was obtained by integrating the area of each temperature point on the fitted curve and dividing it by the total integral area of the curve. This method is similar to that reported in the literature [36,37]. The α-T curves in Figure 10 describe the variations in the conversion degree (α) of four materials with changes in temperature. The Ea-α curves in Figure 11 were obtained by the Friedman method. It can be seen from the Ea-α curves that the activation energy of RDX+ZIF-67 is much lower than that of the other three composites. The Ea value of RDX+ZIF-67 decreases when α rises from 0 to 0.4, but it shows an upward trend as α continues to increase to 1. This indicates that autocatalytic reactions take place in the early stage, resulting in the decrease in the activation energy with the deepening reaction. The HMX+ZIF-67 curve shows a slow decline trend when α increases from 0 to 1, demonstrating minor autocatalytic activities. The activation energy of CL-20+ZIF-67 changes little and keeps stable as the reaction advances. The activation energy of DAP-4+ZIF-67 elevates continuously during the whole reaction process, suggesting that the catalyst plays a greater catalytic role in the initial stage. As the reaction proceeds, its catalyzing effect weakens, and its reaction energy barrier increases.

**Figure 12:**
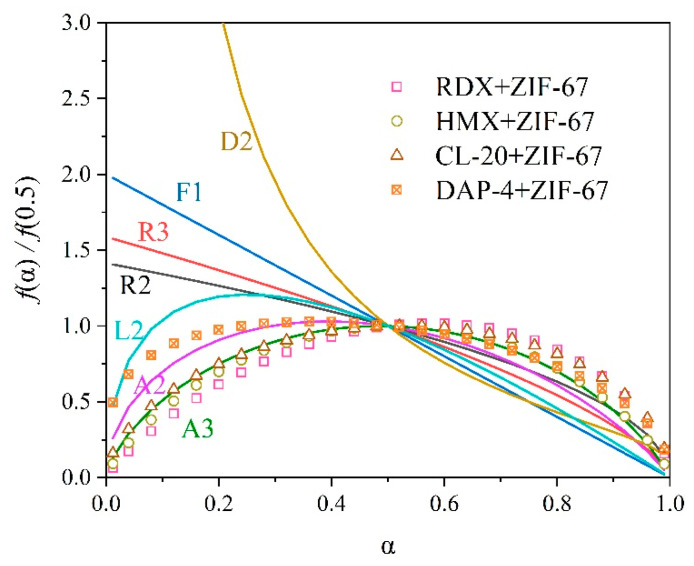
Dynamic model curves.

The kinetic model curves for the RDX+ZIF-67, HMX+ZIF-67, CL-20+ZIF-67 and DAP-4+ZIF-67 composites under the catalysis of ZIF-67 were determined in this paper by the combined kinetic method. The standard models are shown in Figure 12. D2 is a two-dimensional diffusion model. L2 is a random chain fracture model. R3 is a phase interface control model. F1 is a first-order chemical reaction model (single molecule degradation law). A2 and A3 are random two-dimensional or three-dimensional nucleation/growth models [38]. The curves of RDX+ZIF-67, HMX+ZIF-67, and CL-20+ZIF-67 resemble the A3 random three-dimensional nucleation model, and the DAP-4+ZIF-67 curve resemble the A2 random two-dimensional nucleation model. Under the action of the catalyst, the curves of four energetic materials deviated from the standard models to varying degrees. Among them, CL-20+ZIF-67 had the smallest deviation and RDX+ZIF-67 had the largest deviation.

## 3. Materials and Methods

### 3.1. Materials

ZIF-67 was synthesized and prepared according to the method in 3.2.1, RDX (Class 5), HMX (120 mesh), CL-20 (Class 5, 121~180 μm), DAP-4 (50~90 μm) and AP (Class 3, 90~140 μm).

### 3.2. Sample Preparation

#### 3.2.1. ZIF-67 Synthesis Preparation

Co(NO_3_)_2_·6H_2_O (1 mmol, 249.0 mg) and 2-methylimidazole (4 mmol, 328.0 mg) were dissolved in 25 mL ethanol separately. After stirring and mixing the two solutions at 25 °C for 0.5 h, the resultant solution was precipitated for 24 h. Finally, the precipitate was centrifuged to separate the washing product. The separation yield was 86%.

When increasing the feed amount of the materials by 10 times and preparing ZIF-67 in the same reaction conditions, the separation yield was 78%.

In case of poor dispersibility during the preparation process, the materials were fully stirred or ultrasonically treated until they were completely dispersed in the solution.

#### 3.2.2. Mixed Preparation of ZIF-67 and Energetic Materials

An amount of 2 mg ZIF-67, 98 mg RDX, HMX, CL-20, DAP-4 or AP was weighed, then ZIF-67 with a weight of 2mg and a kind of energetic material with a weight of 98 mg were put into the grinding bowl and ground for more than 30 minutes to mix evenly.

### 3.3. Characterization

The thermal behavior was performed with a differential scanning calorimeter (DSC; Netzsch STA 449 F3).

The mass spectra (MS) were obtained with a QMS403 Four Bar Mass Spectrometer: test quality range, 1–300 amu; resolution < 0.5 amu; detection limit > 1 ppm.

## 4. Conclusions

The development of lead-free green EMOF-based energetic burning rate catalysts that can catalyze various solid propellant components has always been an important and challenging topic in the field of solid propellants. ZIF-67 has a large specific surface area and excellent thermal stability. Its catalytic effects on RDX, HMX, CL-20, AP and DAP-4 are investigated in this study. The first four substances listed are the main energetic components of solid propellants, and the last one mentioned is a new heat-resistant energetic material. From our research, the following conclusions can be drawn:

With a decomposition peak temperature above 450 °C, ZIF-67 does not interfere with the maximum thermal decomposition peak temperature of most energetic materials and has high thermal stability. Moreover, ZIF-67 has low mechanical sensitivity, with an impact sensitivity > 40 J and friction sensitivity > 360 N. Thus, it is good and safe to use.

A quantity of 2 wt% of ZIF-67 had excellent catalytic effects on five compounds. ZIF-67 reduced the thermal decomposition peak temperatures of RDX, HMX, CL-20 and DAP-4 by 22.3 °C, 18.8 °C, 4.7 °C and 10.5 °C, respectively. Additionally, ZIF-67 lowered the low- and high-temperature thermal decomposition peak temperatures of AP by 27.1 °C and 82.3 °C, respectively.After adding the burning rate catalyst, the heat release of HMX can be increased from 1173 J·g^−1^ to even 1564 J·g^−1^, which also indicates that ZIF-67, as a general burning rate catalyst, has a very excellent catalytic effect on HMX.

The MS result shows that ZIF-67 has a catalytic effect on HMX, catalyzes the exchange of nitro and nitroso, and causes the start temperature of NO release in the early stage of decomposition to be greatly advanced. ZIF-67 also has a catalytic effect on the splitting of the HMX ring; after adding ZIF-67, the catalysis promotes the formation of CH_2_O and N_2_O during the decomposition of HMX and inhibits the formation of HCN and NO_2_.The kinetic parameters of four compounds, namely, RDX+ZIF-67, HMX+ZIF-67, CL-20+ZIF-67 and DAP-4+ZIF-67, were investigated by Kissinger, Friedman isoconversional and combined kinetic methods. After the addition of ZIF-67, the activation energy of the four energetic materials decreased to varying degrees. Notably, the activation energy of HMX+ZIF-67 dropped by approximately 190 kJ·mol^−1^ when compared with that reported for HMX in previous studies.The dynamic model curves for different composites were determined by the combined kinetic method. The model curves of RDX+ZIF-67, HMX+ZIF-67, and CL-20+ZIF-67 were found to fit the A3 random three-dimensional nucleation model, and the DAP-4+ZIF-67 curve conformed to the A2 random two-dimensional nucleation model.

The catalytic mechanism of ZIF-67 on energetic materials was revealed. Firstly, different interactions between ZIF-67-added organic ligands and metal centers affected the structural evolution of the catalyst during the combustion catalytic reaction and thereby improved the catalytic efficiency. Secondly, with a regular pore structure and a large specific surface area, ZIF-67-based EMOFs could in close contact with energetic components. Thirdly, on account of the regular pore structure, the particle size was distributed uniformly in ZIF-67-added EMOFs, which was able to more easily alter the decomposition process of energetic materials in the sub surface reaction zone or on the combustion surface. ZIF-67-added EMOFs exerted synergistic and homogenous catalytic effects at the molecular level and notably accelerated the thermal decomposition rate. Fourthly, ZIF-67-added EMOFs contain a moderate amount of carbon, so they act as a catalytic bed to enrich metal cobalt and prevent cobalt condensation.

## Figures and Tables

**Table 1:** Kinetic parameters calculated by Kissinger, Friedman isoconversional and combined kinetic methods.

Sample	Kissinger Method			Friedman Method		Combined Kinetic Method		
	*E*_a(1)_	logA	r	*E*_a(2)_	r	m	n	*E*_a (3)_
RDX+ZIF-67	172.47	12.68	0.999	85.65	0.998	0.852	0.621	85.14
HMX+ZIF-67	190.52	12.75	0.998	196.15	0.996	0.778	0.755	182.88
CL-20+ZIF-67	179.08	12.22	0.999	192.92	0.998	0.587	0.523	193.14
DAP-4+ZIF-67	151.43	5.99	0.974	212.69	0.983	0.276	0.479	211.74

*E*_a_: activation energy, kJ·mol^−1^; logA: logarithm of the pre-exponential factor, min^−1^; r: correlation coefficient; m and n are the parameters for the SB function of combined kinetic method.

## Data Availability

Not applicable.
